# Pepper-pot Skull in a Young Male Patient

**DOI:** 10.4274/balkanmedj.galenos.2020.2020.4.125

**Published:** 2020-10-23

**Authors:** Ankur Jain, Praveen Sharma

**Affiliations:** 1 Clinic of Hematology, Vardhman Mahavir Medical College & Safdarjung Hospital, New Delhi, India; 2 Clinic of Hematology, Postgraduate Institute of Medical Training and Research (PGIMER), Chandigarh, India

A 17-year-old boy experiencing fever and bone pain was admitted for a duration of two weeks in our hospital. Examination revealed pallor and diffuse tenderness over sternum, ribs and vertebral bodies. There was no lymphadenopathy or hepato-splenomegaly. Complete blood count were: hemoglobin-80 g/l, white cell count-5.6x10^9^/l, differential counts- 85% neutrophils, 10% lymphocytes, 3% eosinophils, 2% monocytes and platelets-150x10^9^/l. Biochemical analysis revealed: blood urea-280 mg/dL, creatinine-4.0 mg/dL, serum calcium (albumin-corrected)-14 mg/dL, phosphorous- 3.5 mg/dL, uric acid-7 mg/dL, potassium-4.5 meq/L, sodium-135 meq/L and lactate dehydrogenase- 1100 U/L. Liver function tests showed normal results. Work-up for hypercalcemia was: Parathyroid hormone (PTH)-6.2 pg/mL, 25 -hydroxy vitamin D-10 ng/mL and 1, 25-dihydroxy vitamin D (calcitriol)- 15 ng/mL. Multiple lytic lesions involving the skull, ribs and vertebral bodies were revealed in the skeletal survey (Figure 1). Serum protein electrophoresis and immunofixation studies failed to identify a monoclonal protein. Bone marrow aspirate (BMA) showed sheets of blasts involving the marrow space (Figure 2). Blasts were negative for myeloperoxidase (MPO), and immunophenotype by flow cytometry was consistent with pre-B cell acute lymphoblastic leukemia (ALL) (positive for CD10, CD19, CD34, HLA-DR, and TdT, negative for CD3, CD5, CD7, and myeloid antigens). Cytogenetic findings were normal, and reverse transcriptase polymerase chain reaction was negative for ETV6-RUNXI, KMT2A-MLL, BCR-ABL, and E2A-PBX1 mRNA transcripts. The kidneys were bilaterally enlarged with preserved cortico-medullary differentiation as revealed by the ultrasonography of the abdomen.

Hypercalcemia was managed initially with normal saline, furosemide, zoledronic acid, and calcitonin, and later by hemodialysis because of the persistent elevation of serum calcium. Induction therapy of ALL with prednisolone, vincristine, daunorubicin, cyclophosphamide, and L-asparaginase (BFM-90 protocol) resulted in normalization of serum calcium and renal functions within 2 weeks, and patient attained a complete morphological remission after 4 weeks. Written and informed consent was obtained from the patient’s father prior for the publication.

The index case reported here presented with hypercalcemia, acute renal failure, anemia, and had osteolytic bone lesions (CRAB), without any circulating blasts. Multiple myeloma and primary hyperparathyroidism were left out in the initial battery of test. The diagnosis of ALL was revealed after BMA. Such a presentation of ALL is unusual, and is limited to few case reports (1,2). Hypercalcemia at the time of initial presentation in ALL (0.6-4.8%), and concomitant osteolytic bone lesions are extremely rare. Such cases are characterized by older age (10-20 years), normal peripheral blood counts with no circulating atypical cells (aleukemic presentation), absence of organomegaly/lymphadenopathy, aberrant myeloid antigen expression on blasts, an association with t(17,19), and carry a similar prognosis as cases without hypercalcemia (3,4,5). In ALL, hypercalcemia can result either as a result of local bone destruction or humoral factors (PTHrP, interleukin-1, interleukin-6, prostaglandin E2, and tumor necrosis factor) released by lymphoblasts and resulting in activation of the osteoclasts (3,4,5). Current case highlights the need to consider ALL in the differential diagnosis of a patient presenting with “CRAB” features even if no blasts are identified in the peripheral blood. BMA is an investigation which is indispensable to establish the etiology for PTH-independent hypercalcemia, and must be performed before starting corticosteroids to avoid missing the diagnosis of ALL.

## Figures and Tables

**Figure 1:**
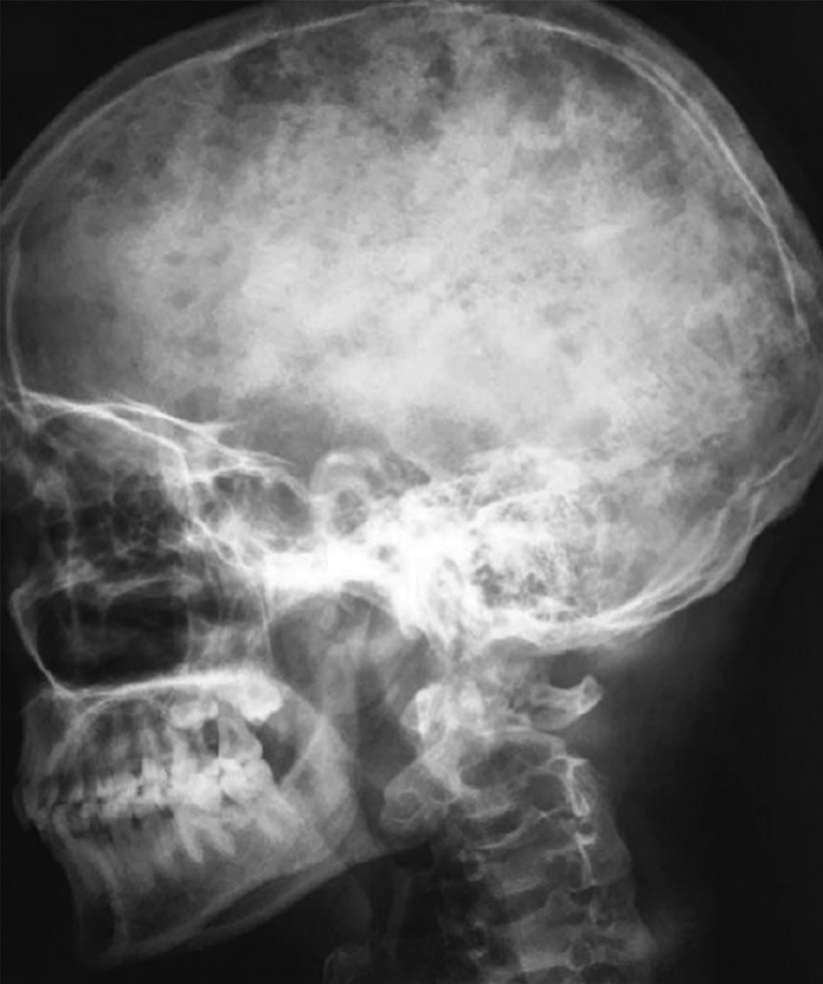
Radiograph of the skull showing multiple osteolytic lesions giving a characteristic “pepper-pot” appearance.

**Figure 2:**
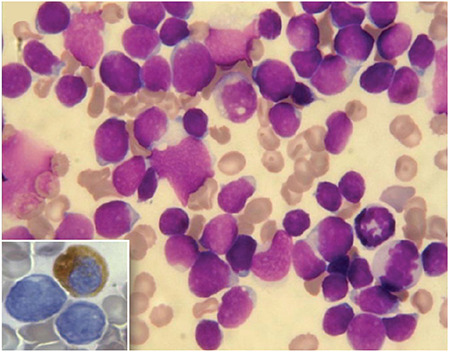
May Grunwald Giemsa stained bone marrow aspirate smear of the patient showing lymphoid looking blasts, which are cytochemically negative for myeloperoxidase stain (inset) (magnification x400).
